# SPECT Imaging of 2-D and 3-D Distributed Sources with Near-Field Coded Aperture Collimation: Computer Simulation and Real Data Validation

**DOI:** 10.1007/s40846-016-0111-6

**Published:** 2016-02-18

**Authors:** Zhiping Mu, Lawrence W. Dobrucki, Yi-Hwa Liu

**Affiliations:** Section of Cardiovascular Medicine, Department of Internal Medicine, Yale University School of Medicine, New Haven, CT 06520 USA

**Keywords:** Near field, Coded aperture, Single-photon emission computerized tomography (SPECT), Maximum likelihood expectation maximization (MLEM), Ordered subset expectation maximization (OSEM)

## Abstract

The imaging of distributed sources with near-field coded aperture (CA) remains extremely challenging and is broadly considered unsuitable for single-photon emission computerized tomography (SPECT). This study proposes a novel CA SPECT reconstruction approach and evaluates the feasibilities of imaging and reconstructing distributed hot sources and cold lesions using near-field CA collimation and iterative image reconstruction. Computer simulations were designed to compare CA and pinhole collimations in two-dimensional radionuclide imaging. Digital phantoms were created and CA images of the phantoms were reconstructed using maximum likelihood expectation maximization (MLEM). Errors and the contrast-to-noise ratio (CNR) were calculated and image resolution was evaluated. An ex vivo rat heart with myocardial infarction was imaged using a micro-SPECT system equipped with a custom-made CA module and a commercial 5-pinhole collimator. Rat CA images were reconstructed via the three-dimensional (3-D) MLEM algorithm developed for CA SPECT with and without correction for a large projection angle, and 5-pinhole images were reconstructed using the commercial software provided by the SPECT system. Phantom images of CA were markedly improved in terms of image quality, quantitative root-mean-squared error, and CNR, as compared to pinhole images. CA and pinhole images yielded similar image resolution, while CA collimation resulted in fewer noise artifacts. CA and pinhole images of the rat heart were well reconstructed and the myocardial perfusion defects could be clearly discerned from 3-D CA and 5-pinhole SPECT images, whereas 5-pinhole SPECT images suffered from severe noise artifacts. Image contrast of CA SPECT was further improved after correction for the large projection angle used in the rat heart imaging. The computer simulations and small-animal imaging study presented herein indicate that the proposed 3-D CA SPECT imaging and reconstruction approaches worked reasonably well, demonstrating the feasibilities of achieving high sensitivity and high resolution SPECT using near-field CA collimation.

## Introduction

Single-photon emission computerized tomography (SPECT) is a non-invasive imaging modality that has been widely used over three decades in nuclear cardiac imaging for the detection of coronary artery disease. Cardiac SPECT is also a molecular imaging modality that allows the visualization of particular molecular processes, such as angiogenesis [1–4] and apoptosis [5, 6], in the heart. Each of these molecular processes can be evaluated via SPECT imaging of a specific biomarker labeled with a molecularly targeted radiotracer [2–4]. Nonetheless, image quality is paramount in molecularly targeted SPECT imaging because the contrast in the target region (concentration of the radiotracer) can be low and embedded in strong background activities, and thus a high-sensitivity and high-resolution imaging technique is required to distinguish the target from the background. As common in other imaging techniques, sensitivity and resolution dictate SPECT image quality [7, 8]. Sensitivity is reflected in photon counts recorded by the system, which in turn determines the signal-to-noise ratio (SNR) of the image. Resolution measures the fine details that can be discerned in the image, representing the clarity of spatial information, which is crucial in various medical applications. As a molecular imaging technique of choice, SPECT also faces challenges arising from the conflicting requirements of high sensitivity and high resolution. Since there is no predefined source-detector geometry (contrary to transmission imaging modalities such as X-ray computed tomography) or the coincidence of a pair of photons that travel in opposite directions, as in positron emission tomography, collimation is required for SPECT imaging and selection of the collimation essentially determines the sensitivity and resolution of SPECT systems. Among conventional methods, high-resolution collimations (e.g., pinhole) often yield low sensitivity, while high-sensitivity collimations (e.g., parallel-hole) generate images with poor resolution. To accommodate both, coded aperture (CA) collimation has been proposed as a potential method to achieve reasonably high sensitivity and high resolution in SPECT imaging [9–14]. This collimation technique was originally devised for astronomical imaging, where incoming projections from a given source (such as a star) are parallel [15–17]. The decoding method for recovering the source distribution, from a linear system point of view, is a direct inverse filtering process that may yield an ideal solution if there is no noise in the recorded image. If noise is present, however, in particular when the noise is white or wide-band, as is the case in most medical images, such a simple inverse filtering process leads to severe artifacts, such as negative values in the decoded image, due to noise amplification [13, 18, 19]. Traditional CA imaging and decoding techniques do not work well in three-dimensional (3-D) near-field applications such as in small-animal imaging, and thus only limited success has been achieved with two-dimensional (2-D) planar imaging [17–19]. The main impediment of near-field CA imaging appears to be the multiplicity of possible paths for each incoming source and the complexity of the diverging projections from sources at different depths.

In previous studies [9, 11, 12], it was discovered that the angular artifacts due to the aperture collimation effect of CA imaging is mostly neglected by researchers in the field. A solution has been proposed to correct for this collimation effect [12]. An iterative algorithm, a convolution form of the maximum likelihood expectation and maximization (MLEM) algorithm, was applied in lieu of the conventional correlation-based decoding method for image reconstruction, which offered certain desirable radionuclide image characteristics, including preservation of total counts and non-negativity of pixel values in the reconstructed images [12]. Previous studies have also proposed processing the entire recorded image and suggested the use of a single mask pattern instead of the commonly used mosaic pattern [13, 17–19]. Incorporating these approaches into a series of phantom studies, a previous study obtained 2-D reconstructions of phantom images with remarkably improved image quality [12]. Furthermore, a quasi-3-D image reconstruction algorithm has been proposed to restore an image stack from a single CA projection and its capability of discerning two planar objects located at different depths has been verified [11]. More recently, a multi-angle imaging protocol was employed and an image reconstruction method was proposed for true 3-D near-field CA SPECT that integrates the quasi-3-D reconstruction algorithm with the ordered subset expectation maximization (OSEM) algorithm [20]. With the preliminary success of attempts to reconstruct true 3-D near-field CA images, previous studies have achieved reasonable SPECT reconstructions of 3-D objects, including a micro hot-rod phantom with complex structures [11, 12].

While these previous results were encouraging, the objects imaged were simply comprised of fillable rods or capillary tubes with strong positive contrast (hot-spot) and sharp edges [9, 11, 12]. Some early studies have concluded that CA provides higher SNR than that of pinhole only in the condition of bright (i.e., hot-spot) objects positioned within a small field of view [13, 21, 22]. These studies employed the uniformly redundant array (URA) decoding method [15, 16], which is a linear process for image recovery. Recently, significant progress has been made in a variety of image reconstruction applications, and nonlinear methods, such as the MLEM algorithm [9, 11, 12], have been shown to provide superior results. Hence, it would be of great interest to investigate the performance of 3-D CA SPECT reconstruction methods for objects with low or negative contrast (cold-spot) and a slowly varying radioactivity distribution, which are common in pre-clinical (small animal) and clinical SPECT imaging of myocardial perfusion deficiencies.

In this study, we first corroborate the superiority of CA over single-pinhole collimation in imaging distributed sources with both positive and negative contrast as well as over multi-pinhole collimation in imaging distributed sources of various resolutions using computer simulations. We then introduce a unique scheme of 3-D near-field CA SPECT reconstruction and demonstrate the feasibilities of the proposed methods to reconstruct low, negative-contrast, and slowly varying radioactivity distribution in an ex vivo rat heart post myocardial infarction (MI).

## Materials and Methods

### Computer Simulation of Distributed Sources

**Fig. 1:**
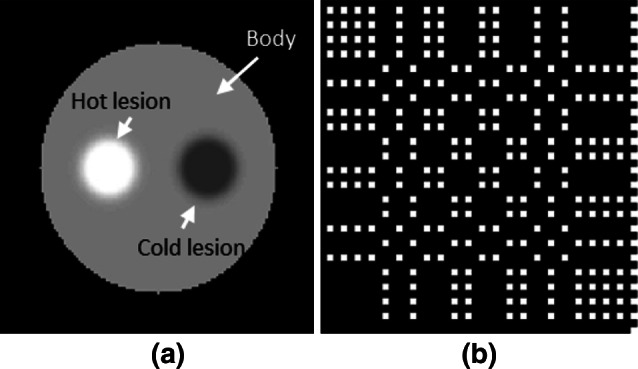
Digital phantom and CA mask. **a** Phantom image with relative activities in body and hot and cold lesion regions set equal to 1, 1.5, and 0.5, respectively and **b** CA mask with 46 × 46 NTHT MURA

We designed a series of computer simulations with objects that were previously deemed unfavorable for CA imaging [21] and compared the CA results with those of single-pinhole images. A round-shaped digital phantom with a hot lesion and a cold lesion (Fig. 1a) was used in the computer simulations. The relative activity was 1.0 in the body of the phantom, 1.5 in the hot lesion, and 0.5 in the cold lesion. A single (not mosaic) 46 × 46 no-two-holes-touching (NTHT) modified uniformly redundant array (MURA), shown in Fig. 1b, was used for the CA mask. The hole in the pinhole collimator (not shown) was of the same size as the holes in the CA mask. In this configuration, a magnification of 1 was used for both pinhole and CA images. Hence, the pinhole images were of the original size, and the CA projection was the result of the convolution of the digital phantom and the mask shown in Fig. 1b. Multiple simulations were performed with a series of base radioactivities (3, 10, 30, and 100). A low detector noise of 0.1 was added to each of the radioactivity levels simulated. For example, in the simulation with a base activity of 10, the pixel values were 10 for the body background, and 15 and 5, respectively, for the hot and cold lesions simulated in the phantom. The pinhole image was generated from the Poisson distribution, with the phantom image as the mean, i.e., each pixel value was a Poisson random number with the mean equal to the corresponding phantom pixel value plus the detector noise. Similarly, the CA projection was generated from a Poisson distribution with the convolution of the phantom and the mask plus the detector noise as the mean. The CA images were reconstructed using the MLEM algorithm described herein and compared with the pinhole images.

### Computer Simulation of Distributed Sources with Various Spatial Resolutions

**Fig. 2:**
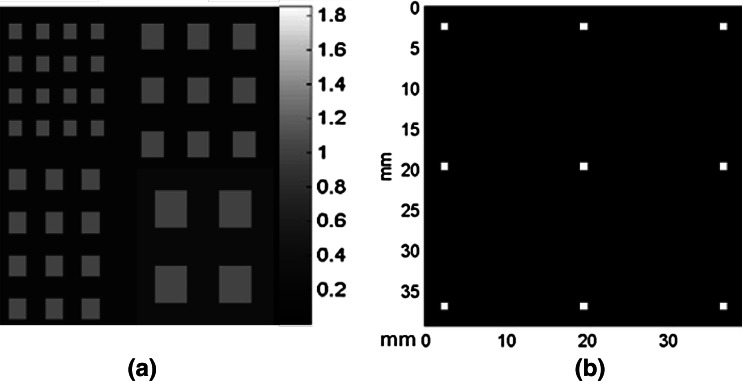
Digital resolution phantom and 9-pinhole collimator. **a** Computer-simulated resolution phantom with four groups of rods of equal activities. Background activities were added, as 30% of rod activities at lower right quadrant, and 20% elsewhere. **b** Computer-simulated 9-pinhole collimator with 1-mm aperture in each pinhole and 1.75-cm distance between pinholes

**Fig. 3:**
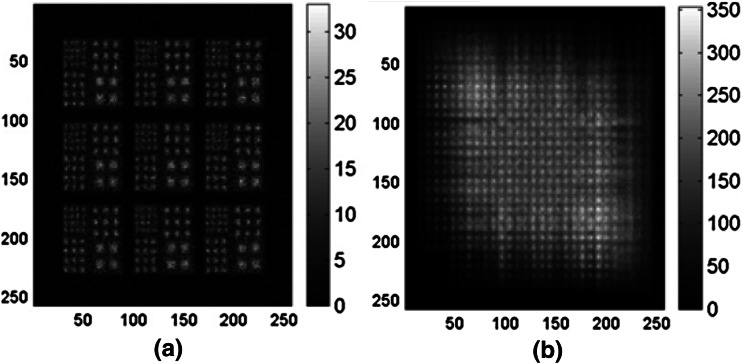
Computer-simulated projections with added Poisson noise. **a** Simulated 9-pinhole collimated images of digital resolution phantom shown in Fig. 2a and **b** simulated CA collimated image of the same digital phantom

We further designed a computer simulation with a digital resolution phantom comprising four groups of squared hot rods with equal activities, as shown in Fig. 2a. The rod sizes were 1.0, 1.33, 1.67, and 2.33 mm (i.e., 3, 4, 5, and 7 pixels), respectively, and rod center-to-center separations were twice the rod sizes. A computer-simulated 9-pinhole collimator comprising 9 pinholes arranged in a 3 × 3 format with equal spacing, as shown in Fig. 2b, and the CA mask shown in Fig. 1b were used in this simulation. We particularly chose the 9-pinhole collimator for comparison with CA because 9 has been accepted by other investigators as the optimal number of pinholes for small-animal brain imaging [23]. The CA mask is an extreme form of multi-pinhole collimation with severe multiplexing of photon paths, which has been deemed detrimental to image quality due to increased complexity of the recorded image. The resolution phantom shown in Fig. 2a was convolved with the 9-pinhole collimator and CA mask to generate a 9-pinhole and CA projections with the same magnification factor. The size of the software phantom, the spacing between the pinholes on the 9-pinhole collimator, and the magnification factor were chosen such that the projected images were of approximately the same size. There was no overlap or truncation in the 9-pinhole projection, as shown in Fig. 3a. Poisson noise was added to both the 9-pinhole and CA projections. The total numbers of counts simulated in the pinhole (Fig. 3a) and CA projections (Fig. 3b) were 266,634 and 5,988,960, respectively. Both projections were reconstructed via the MLEM algorithm described below.

### MLEM Image Reconstruction

The MLEM algorithm used for reconstructing the radioactive emissions was derived from the Poisson noise model and maximization of *P*(*s|p*), the probability of an estimated image *s* given the recorded image *p*. A negative pixel value is not permitted in the Poisson model, as in reality negative radioactivity does not exist. Therefore, this method ensures the non-negativity of the reconstructed image, a highly desired advantage over the conventional URA decoding method. Other benefits of the MLEM algorithm include improved contrast-to-noise ratio (CNR), elimination of the need for a mosaic mask pattern [15, 16, 21], and suppression of noise background outside the object. Another property of the iterative MLEM algorithm is its nonlinear operation, which makes it fundamentally different from other methods, in which derivations and analyses were built on the linear model described by Fenimore [21]. Interested readers may refer to a previous study [12] for more details about this MLEM approach.

### Near-Field Coded Aperture SPECT Imaging of Ex Vivo Rat Heart

A small-animal study was conducted to evaluate the capability of imaging cold-spot lesions in the heart. A male Lewis rat (400 g) was anesthetized by an intraperitoneal injection of ketamine (60 mg/kg) and xylazine (10 mg/kg), intubated, and mechanically ventilated with 1% isofluorane and 99% oxygen for the duration of an open-chest surgery. A left thoracotomy was performed in the fourth intercostal space, and after opening the pericardium, the left anterior descending (LAD) coronary artery was ligated with a 6-0 prolene suture at ~7 mm below the origin to induce permanent MI in the anterior apical region of the left ventricular (LV) myocardium, resulting in significant reduction of myocardial perfusion. The chest was closed in layers after the surgical procedures and then 2.33 × 10^8^ Bq (6.29 mCi) of ^99m^Tc-Tetrofosmin (photo peak: 140 keV) was injected into the jugular vein. One hour after the radiotracer injection, the rat was sacrificed and the LV was dissected from the heart. Dental alginate was subsequently injected into the LV to sustain its shape for imaging. The dissected heart exhibited a cold-spot lesion embedded in the “hot” regions, in which the myocardial perfusion was relatively normal. This activity distribution was slow-varying in nature, making it a good model for testing the imaging capability of the proposed imaging technique.

**Fig. 4:**
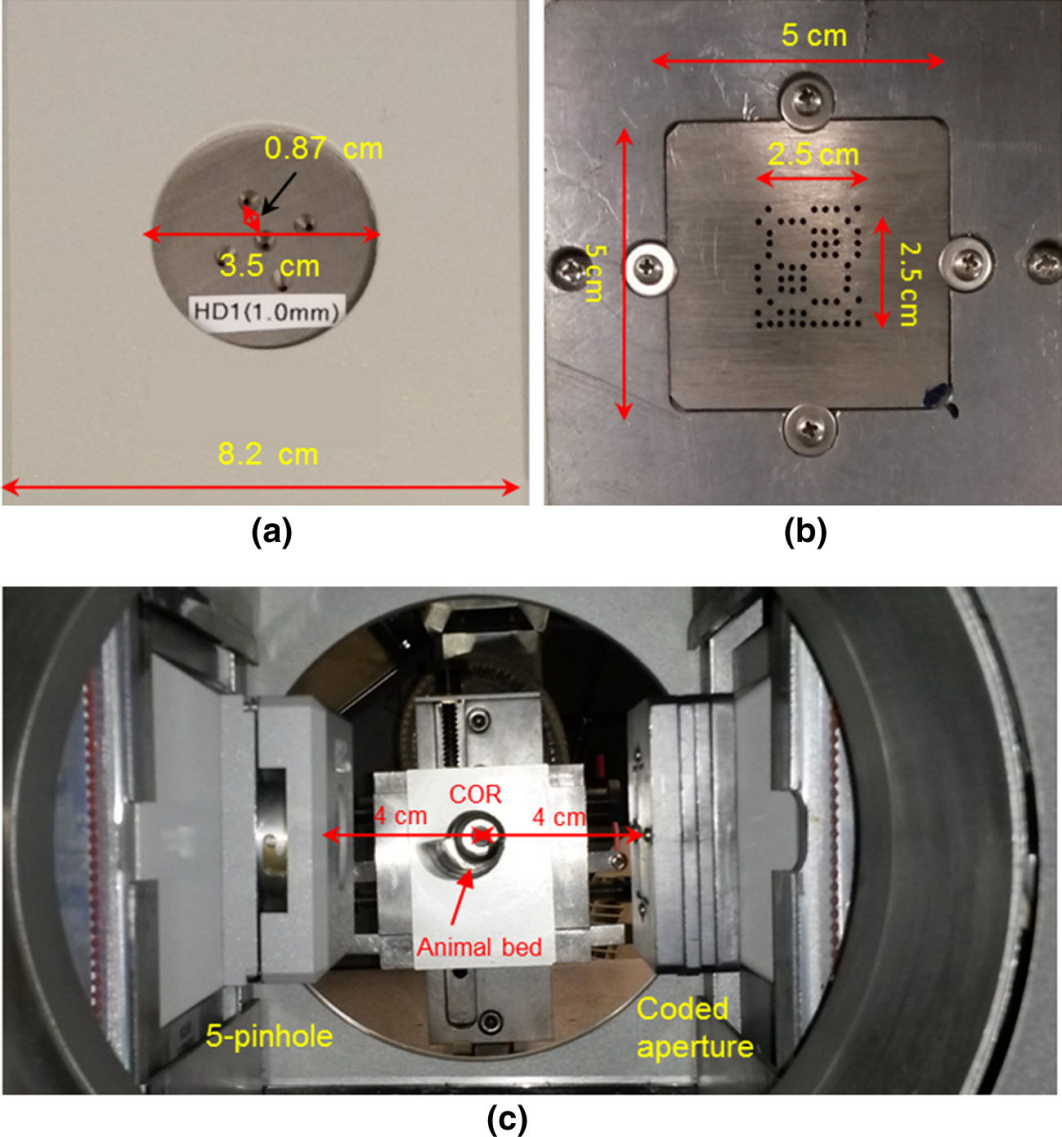
CA collimator module used for imaging of rat heart. **a** Commercial 5-pinhole collimator, **b** custom-manufactured CA mask with 11 × 11 NTHT MURA, and **c** circular micro-SPECT camera gantry equipped with 5-pinhole collimator and CA module

The rat heart was imaged using a dual-headed micro-SPECT system (X-SPECT, Gamma Medica-Ideas, Inc., Northridge, CA), with one head mounted with a 5-pinhole collimator (Fig. 4a) and the other with a CA module (Fig. 4b). The commercial pinhole collimator shown in Fig. 4a consisted of five pinholes configured in a special circular geometry with a radius of 3.5 cm to minimize the overlap of projections. This collimator had four tilted peripheral pinholes and one non-tilted pinhole located in the center of the collimator. All of these pinholes were equipped with 1-mm apertures. The tilt angles were 14° for the peripheral pinholes, which were all focused to an external point ~4 cm away from the center of the aperture plate. The size of the collimator was 8.2 cm × 8.2 cm and the distance between the center pinhole and each peripheral pinhole was 0.87 cm, as shown in Fig. 4a. The CA mask custom-designed for this study was an 11 × 11 NTHT MURA. The aperture area was 2.5 cm × 2.5 cm, with the aperture diameter and mask thickness both equal to 1.0 mm. The size of entire CA mask mounted in the collimator module was 5 cm × 5 cm, as shown in Fig. 4b. The 5-pinhole collimator and CA module were mounted on the dual micro-SPECT camera heads as shown in Fig. 4c. Each of these camera heads was equipped with a sodium iodide (NaI) detector array with a sensitive area of 12 cm × 12 cm and pixilated into an image matrix size of 80 × 80 pixels (1.5 mm × 1.5 mm in pixel size). A total of 128 (i.e., 64 pinhole and 64 CA) projections were acquired evenly over a 360° camera rotation with a center of rotation (COR, i.e., mask to rotation axis distance) of 4 cm and an imaging time of 60 s/projection. With different collimator-to-detector distances (5 cm for pinhole and 6.5 cm for CA), the magnification factors for pinhole and CA were calculated as 2.25 (m_p_ = (4 + 5)/4) and 2.6 (m_c_ = (6.5 + 4)/4), respectively.

**Fig. 5:**
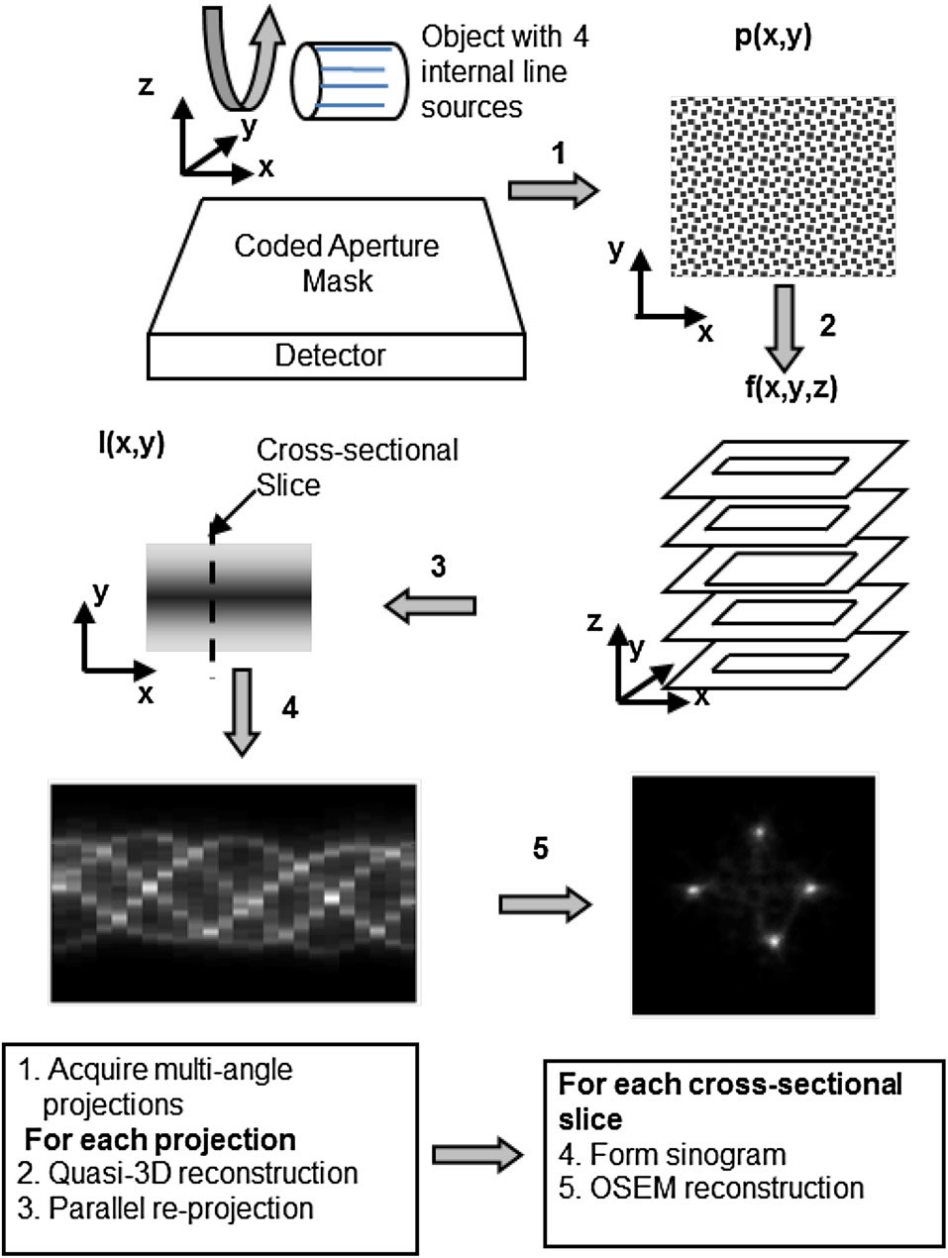
Illustration of near-field CA imaging and 3-D image reconstruction steps

Pinhole images were reconstructed using the commercial SPECT reconstruction software provided by the micro-SPECT system (X-SPECT, Gamma Medica-Ideas, Inc.), whereas CA images were reconstructed via the 3-D CA SPECT reconstruction algorithm illustrated in Fig. 5 [11]. No attenuation or scatter corrections were applied to the image reconstructions. The CA SPECT reconstruction was implemented in five major steps: (1) multi-angle CA projections were acquired; (2) an image stack, *f*(*x, y, z*), consisting of slices parallel to the detector, was generated from each of the CA projections [12]; (3) synthetic projections *I*(*x, y*) were created, following the parallel projection geometry, by re-projecting *f*(*x, y, z*), which had been corrected for varying pixel sizes at different object depths; (4) sinograms of cross-sectional slices perpendicular to the camera rotating axis were formed by extracting the corresponding columns from the synthetic projections; and (5) the OSEM algorithm [11, 20] was employed to reconstruct cross-sectional SPECT slices from the sinograms.

In a previous study, a small-angle projection approximation [12] was made in the algorithm summarized above, and a center collimation factor matrix, *C*_*C*_ (Eq. (17) in [12]), was used to correct for the collimation effect in the projection image. This small-angle approximation is not justified here because the small-animal imaging system used in this study had a wide projection angle due to the small distances from the CA mask to the object as well as to the detector (see Fig. 4c). As such, we extended the algorithm in Step 1 above to account for these angular variations and spatially varying effect as follows. Firstly, an object plane was divided into *n* × *n* small regions, *f*_*i*_(*z*), *i* = 1,…, *n*^2^; secondly, for each region, a center collimation factor for that region was computed by taking the center of the region as the collimation center, *C*_*Ci*_; then, in the re-projection step, the contribution of the current estimated image plane to the projection was calculated, *p*_*r*_(*z*). More specifically, the $\hat{f}^{(K)} (z)*h(z)$ originally formulated in Eq. (22) of [12] was computed as:

1 $$p_{r} (z) = \sum\limits_{i = 1}^{{n^{2} }} {\left[ {\hat{f}_{i}^{(K)} (z)*h(z)} \right] \times C_{Ci} }$$

where $\hat{f}_{i}^{(K)} (z)$ is the *K*th estimate of the *i*th region of the object image at distance *z*, *h*(*z*) is the mask shadow for the object imaged at distance *z*, and * is the convolution operator. Note that *C*_*Ci*_ is a matrix of the same size as that of *p* (the recorded projection), and × denotes element-wise multiplication. As a result, Eq. (24) in [12] was modified as:

2 $$\hat{f}^{(K + 1)} = \frac{{\hat{f}^{(K)} (z)}}{{\sum_{z} h(z)}}\left[ {h(z) \otimes \frac{{p - \sum_{{z \ne z^{\prime } }} p_{r} (z^{\prime } )}}{{p_{r} (z)}}} \right]$$

where ⊗ is the correlation operator. The object plane in this animal study was divided into four regions in a 2 × 2 configuration to investigate the effect of this new approach in dealing with spatially varied situations in the wide-angle projection case.

## Results

### Computer Simulation of Distributed Sources

**Fig. 6:**
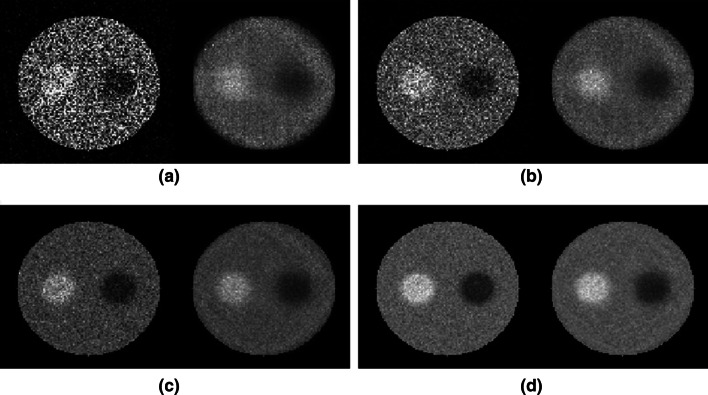
Computer simulation results with four different levels of radioactivity. In each panel of **a**–**d**, reconstructed pinhole image is shown on *left* and reconstructed CA image is on *right*. Base activity = 3, 10, 30, and 100 for (**a**), (**b**), (**c**), and (**d**), respectively

Figure 6 shows comparisons between the pinhole and reconstructed CA images obtained from the computer simulations of distributed sources. As can be seen, the reconstructed CA images were of better quality in all four cases, showing less noise in the body as well as in the lesion regions. The improvement was particularly pronounced in the low-activity cases shown in Figs. 6a, b, where good-quality reconstructions were achieved with the MLEM even in the lowest-activity case (Fig. 6a).

**Fig. 7:**
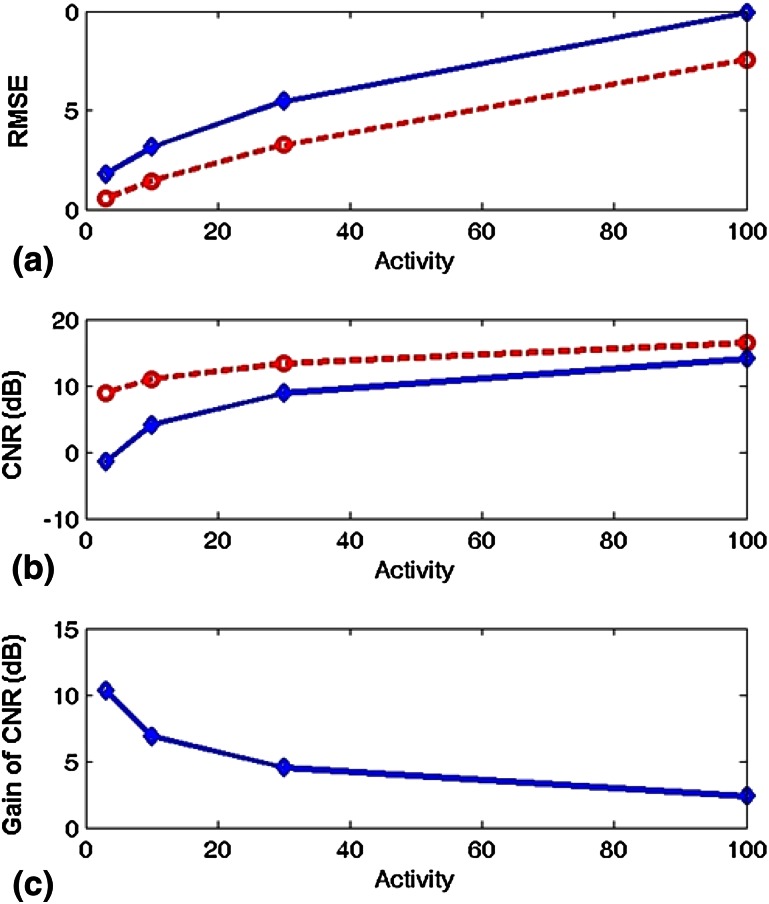
Quantitative comparisons. **a** Plots of RMSE for pinhole images (*solid line*) and reconstructed CA images (*dashed line*) with four different simulated radioactivities, **b** plots of CNR for pinhole images (*solid line*) and reconstructed CA images (*dashed line*), and **c** illustration of CA improvements over pinhole in terms of CNR

Quantitative assessments of image quality are shown in Fig. 7, which confirm the observations in Fig. 6. In all four cases, the reconstructed CA images yielded lower root mean square errors (RMSEs) and higher CNRs than those for the pinhole images. The CNR was calculated as CNR = 20 × log_10_ (0.5 × activity/RMSE) because the hot and cold lesions in the phantom had the same contrast (0.5 × activity). The improvement of CA images as compared to pinhole images was over 10 dB at an activity of 3, and approximately 6.5 and 4.6 dB at activities of 10 and 30, respectively. Even in the high-radioactivity case of 100, the improvement was approximately 2.5 dB. Notice that these improvements respectively represent approximately 90, 78, 65, and 44% reductions on image acquisition time to achieve the given CNRs of 10, 6.5, 4.6, and 2.5 dB, as CNR improves with the square root of the recorded counts based on the Poisson statistics in radionuclide imaging. The time reduction described above was computed as (1 − 10^−x/10^) × 100%, where *x* is the CNR improvement in dB. For instance, the time reduction was calculated as (1 − 10^−0.25^) × 100% = 44% for the CNR improvement of 2.5 dB. To achieve a 10 dB improvement in CNR, one would need to acquire 10 times more counts, and thus the acquisition time would increase 10-fold if the radiotracer dose were fixed. More specifically, the CA technique requires only 1/10 the acquisition time required by the single-pinhole collimation for this case to achieve the same CNR, resulting in a 90% reduction on acquisition time. These results reveal that the CA imaging technology indeed improves the CNR, particularly in low-radioactivity, photon-starved situations, which are often encountered in medical imaging applications. The computer simulations presented above also demonstrate that CA outperforms pinhole collimation for imaging distributed sources with both hot- and cold-spot lesions when the nonlinear MLEM image reconstruction algorithm is employed.

According to Fenimore [21], this phantom is unsuitable for CA imaging. The ratio of the source pixel value to the total source intensity, *Ψ*, was given by *Ψ*_*ij*_ = *S*_*ij*_*/I*_*T*_ in Eq. (17) in Fenimore’s study [21], where *S*_*ij*_ is the pixel value of element *ij* in the source and *I*_*T*_ is the total intensity of the source. In that study [21], it was shown that the improvement of CA over single-pinhole collimation was better for larger *Ψ*, and would be inferior for smaller *Ψ*. The digital phantom used in our study had a large smooth area with constant values; the largest value for *ψ* was only 0.0002 inside the hot lesion. The digital phantom also had a cold lesion area where *ψ* was only 0.00007. According to Eq. (22) in Fenimore’s study [21], the decoded CA image would be inferior to the pinhole image for sources with such small *ψ* values. However, our simulations show the opposite results when the image was reconstructed with the nonlinear MLEM algorithm. Based on our simulation results, we argue that CA imaging with the MLEM reconstruction algorithm mitigates the difficulties in imaging objects with large smooth areas in cold lesions, and, at least in some common situations, it yields reconstructions with higher CNRs than those obtained using pinhole collimation.

### Computer Simulation of Distributed Sources with Various Spatial Resolutions

**Fig. 8:**
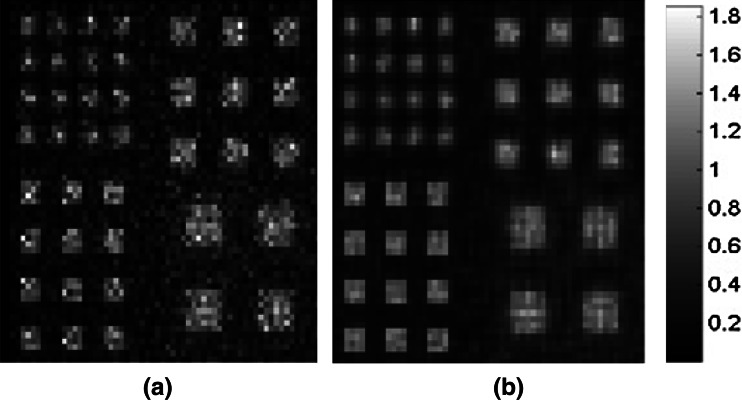
Reconstructed images of digital resolution phantom. Images reconstructed from **a** 9-pinhole collimated projection at 25 MLEM iterations and **b** CA projection at 300 MLEM iterations

**Fig. 9:**
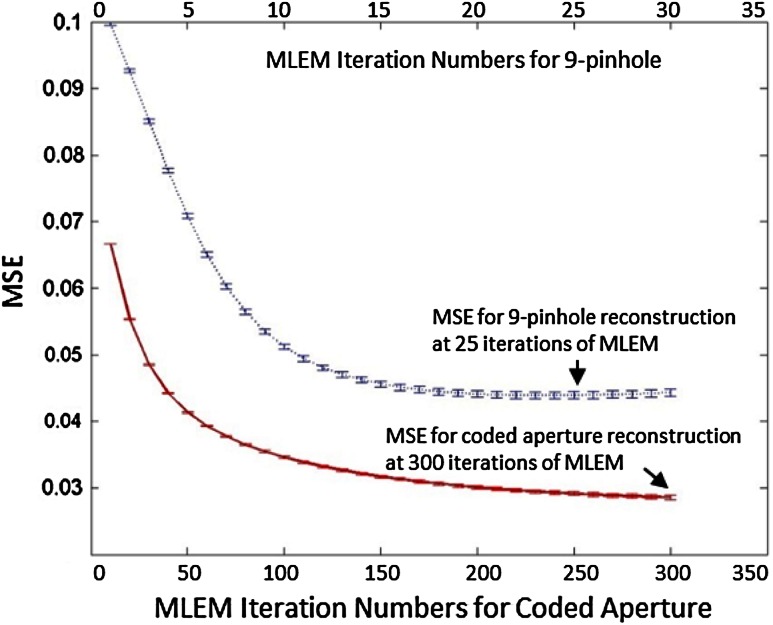
MSE (mean ± SD, n = 10) plots of images reconstructed from 9-pinhole collimator at 1–25 iterations of MLEM (*upper curve*) and from CA at 1–300 iterations of MLEM (*lower curve*)

Figure 8 shows images of the digital resolution phantom reconstructed from the 9-pinhole projection at 25 iterations (Fig. 8a) and from the CA projection at 300 iterations (Fig. 8b) of MLEM reconstructions. As shown, all rods in the phantom can be discerned visually in both sets of reconstructed images, demonstrating that CA collimation resulted in images with resolution similar to that obtained with 9-pinhole collimation. Notice that the 9-pinhole reconstructed image shown in Fig. 8a appears to be noisier than the CA reconstructed image shown in Fig. 8b, presumably due to the low count statistics from pinhole collimation. Images reconstructed from the 9-pinhole projection after 25 iterations of the MLEM are not shown because they were of poorer quality than that shown in Fig. 8a. This resolution phantom simulation was performed 10 times, from which the mean squared errors (MSEs) of image reconstructions at different MLEM iterations were calculated. Figure 9 shows the MSE plots for the pinhole and CA image reconstructions. As shown, the MSEs reach their minimum at the 25th iteration of MLEM for the 9-pinhole collimator and then tend to increase after 25 iterations due to the high noise level (low sensitivity), whereas those for the CA images continue to decrease even after 300 iterations. The MSEs of the images reconstructed from the 9-pinhole collimator, at the lowest level, were about 50% higher than those of images reconstructed from CA at 300 iterations.

**Fig. 10:**
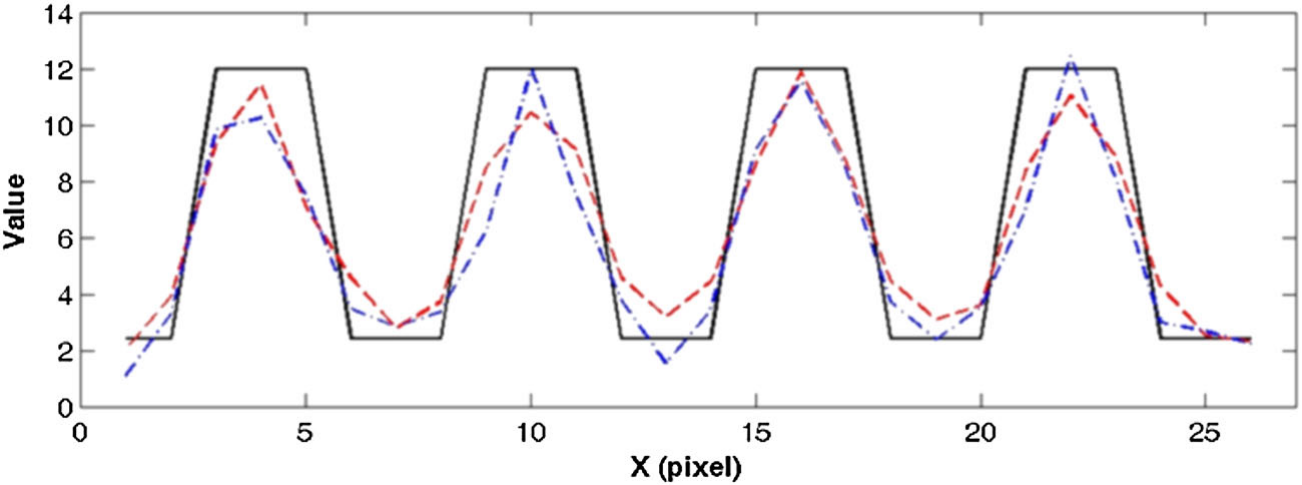
Image profiles of computer-simulated resolution phantoms. Profiles were generated by summing 12 rows (i.e., 3 rows/SROI × 4 SROI) containing the smallest rods in *upper left quadrant of images* shown in the original (Fig. 2a) and reconstructed (Fig. 8) phantom images. Note that profiles were summed to smooth image noise and the smallest SROI in *upper left quadrants* were chosen for profile calculations to demonstrate extreme case of simulated image resolutions. *Solid line* original; *dashed line* CA; *dashed*-*dotted line* 9-pinhole. Profiles from CA and 9-pinhole images are very similar, indicating comparable resolution obtained by these two collimation methods. *SROI* squared region of interest

Figure 10 shows the profiles generated from the smallest rods in the original (Fig. 2a) and reconstructed images (Fig. 8). As shown, the profiles from the CA and 9-pinhole images are very similar, an indication of similar resolution obtained by these two methods.

These computer simulation results also demonstrate that CA images have a resolution similar to that of 9-pinhole images, but show better SNR thanks to the high sensitivity of CA despite its severe multiplexing. Although the 9-pinhole collimator has faster MLEM convergence (Fig. 9), the MLEM process may diverge if the noise level is high.

### Ex Vivo Rat Heart Imaging

**Fig. 11:**
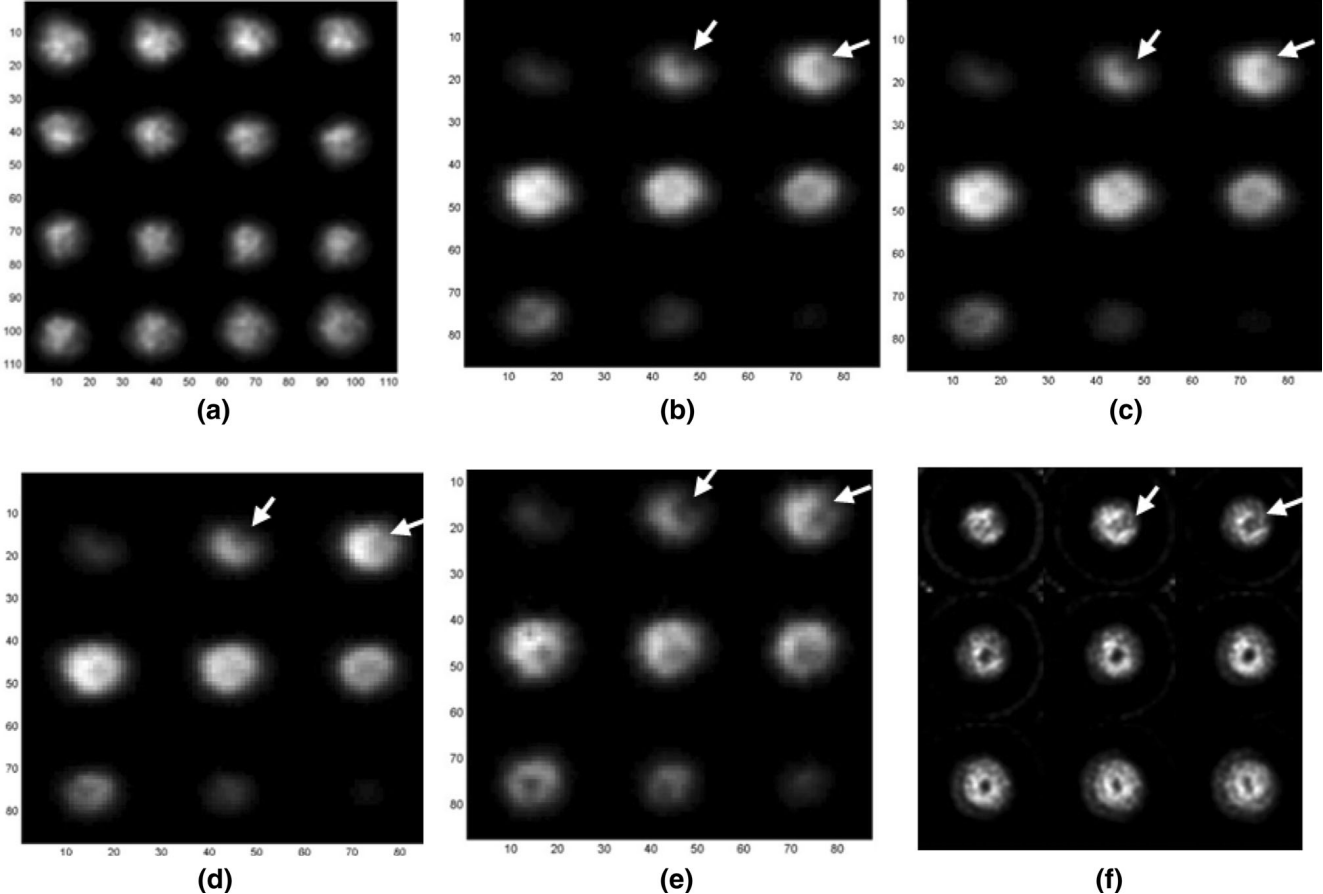
Experimental results of small-animal imaging. **a** Subset of synthetic projections created from image stacks reconstructed using 64 CA images evenly distributed over a 360° camera rotation. Images (pixel size = 0.92 mm) of cross-sectional slices (separated by 1.84 mm) of ex vivo heart respectively reconstructed from **b** 16, **c** 32, and **d** 64 CA projections without accounting for angular differences. **e** Images of the same cross-sectional slices as those shown in (**d**) but with wide-angle correction, resulting in improved image contrast in comparison to those in (**b**–**d**). **f** Images (pixel size = 1.16 mm) of the same rat heart reconstructed from 64 projections of 5-pinhole collimator acquired simultaneously with CA projections. Myocardial perfusion defects in anterior-lateral apical area of LV myocardium caused by MI can be clearly visualized (*arrows*) in (**b**–**e**) as well as in (**f**). Image contrast in **f** appears to be higher than that in (**b**–**e**) thanks to pinhole reconstruction from non-overlapping projections, but noise artifacts are severe in (**f**) due to relatively low count statistics of pinhole

The total counts of 64 SPECT projections acquired simultaneously using the CA and 5-pinhole collimators were 14,631,558 and 602,705, respectively, and thus the sensitivity of CA was 24 times higher than that of the 5-pinhole collimator in this small-animal imaging. Figure 11 shows the results from the small-animal imaging experiment. Figure 11a shows a subset of 16 representative synthetic projections created by re-projecting the image stacks generated from the original 64 CA projections. These images represent projection views of the rat heart as if it were seen through a parallel-hole collimator from 16 angles evenly distributed over a 360° rotation (see Step 3 in Fig. 5). Figure 11b–d show three individual sets of 9 cross-sectional slices reconstructed from 16, 32, and 64 angles of the synthetic projections using 5 iterations and 2, 4, and 8 subsets of the OSEM algorithm [20], respectively, which were then smoothed by a Gaussian filter defined as exp(−(x^2^ + y^2^)/σ^2^), where σ = 0.8 pixels. The reconstructed images (pixel size = 0.92 mm) in each set were separated by 1.84 mm (i.e., with one slice in between) and placed in order from left to right and then top to bottom to display the anatomical sequence of the LV from the apical to basal slices. Notice that the angular differences mentioned above were not accounted for in Step 1 of Fig. 5 for these images, while the donut shape (~1.8 cm in diameter) of the LV was reconstructed reasonably well, even with a small number (16 or 32) of projections. The surgically induced MI, resulting in a myocardial perfusion defect in the anterior-lateral apical regions of the LV myocardium (arrows), can be clearly identified, especially in the slices shown in the top row of Fig. 11b–d. This demonstrates that cross-sectional images can also be reconstructed from a smaller number (e.g., 32 or 16) of projections, with slightly lower but reasonably good contrast. The ability to reconstruct images with good quality using a small number of projections is highly desirable because it shortens the image acquisition time, reduces the required dose of radiotracer injected into the subject, or both. Figure 11e shows images of the cross-sectional slices shown in Fig. 11d but reconstructed with the angular difference correction (Eq. (1)). As can be seen, the images exhibit higher contrast as compared to that of those shown in Fig. 11b–d thanks to the wide-angle correction (Eqs. (1) and (2)) [12]. The method used to account for the large projection angle was rather simple. However, using only the 2 × 2 division, it led to a marked improvement in the image contrast. Further investigation may be needed in this area. Figure 11f shows nine consecutive cross-sectional slices of the same rat heart reconstructed from the 5-pinhole projections using the commercial OSEM software of the micro-SPECT system (X-SPECT, Gamma Medica-Ideas, Inc.) with 8 subsets and 5 iterations. The pinhole SPECT images were also post-filtered by the same Gaussian filter applied to the CA reconstructed image. As expected, the pinhole SPECT images (pixel size = 1.16 mm) show better contrast, particularly in the LV cavity thanks to the non-overlapping pinhole projections, but are noisier than the CA SPECT images shown in Fig. 11e in part due to the low count statistics of the 5-pinhole collimation.

## Discussion

In reviewing the literature relevant to CA imaging, we recognized that previous studies [13, 14, 17–19, 21, 22] mostly utilized a decoding method to recover the original images, a technique that is in fact inverse filtering. Considering that new and more sophisticated data processing methods have been proposed over the years, we believe that the conclusions previously made that were unfavorable for CA imaging [21] may not be applicable to the CA imaging technology currently under development. The derivations and analyses that were built on the linear deterministic model [21] should not be adopted without validation when the images are reconstructed via the nonlinear probabilistic MLEM algorithm. Some of our main arguments and comments are further elaborated below, and our contribution to the field and the limitations of this study are also described.

### Traditional Coded Aperture Imaging and Conventional Decoding Scheme

Traditional CA imaging involves several components: (1) selection of a CA pattern (often a URA or MURA), *f*, with a matching decoding pattern, *g*, so that their correlation is a δ function; (2) creation of a mosaic mask with four identical basic patterns, and use of only the central quadrant of the projected image, *p*, for further processing to ensure the cyclic convolution model of image formation; (3) computation of the decoded image, *s*, by *s* = *p* ⊗ *g*, where ⊗ represents the correlation operation. This traditional imaging method was proposed by Fenimore [21], who gave several important conclusions. One of the most-cited conclusions is that CA performs better in imaging bright point-like sources than distributed sources. It was also suggested [21] that CA is unsuited for imaging cold lesions. A simple explanation of these observations can be articulated as follows. The decoding mask, *g*, consists of both positive and negative elements and the projected image, *p*, consists of only positive elements. In the decoding equation, for any reconstructed pixel *i*, the pixel value *s*_*i*_ can be represented as *s*_*i*_ =*P*_*i*_ − *N*_*i*_, where *P*_*i*_ and *N*_*i*_ are the contributions from positive and negative elements in *g*, respectively. For low-intensity pixels in the original image, *P*_*i*_ and *N*_*i*_ are roughly equal and mostly cancel each other out in *s*_*i*_, while their contributions to noise add up. As a result, low-intensity pixels have low SNR and even negative values in the decoded image. On the other hand, for pixels in bright point-like sources, *P*_*i*_ ≫ *N*_*i*_, and hence they have much higher SNR.

### Coded Aperture SPECT Reconstruction Via MLEM Algorithm

The MLEM algorithm is built on the Poisson statistics of photon emission and detection processes. It is a nonlinear method that is fundamentally different from the decoding methods built on the linear and deterministic model, as described above. As evidenced in the computer simulations and small-animal study reported herein, CA SPECT performed better than expected when the MLEM algorithm was employed in the image reconstruction.

### Contributions and Limitations of Present Study

In the current study, we demonstrated via computer simulations that 2-D CA imaging results in better image quality and higher CNR than those obtained with pinhole imaging. Note that these computer computations were not primarily focused on resolution comparisons but were attempted to show that, in certain cases with limited counts, CA provides results with similar resolution yet higher CNR. This is highly desirable because SPECT imaging is generally operated in photon-starved conditions, and conventional collimations have difficulties in achieving high sensitivity and high resolution simultaneously. Nevertheless, further studies may be warranted to provide thorough comparisons between the pinhole and CA collimations in terms of image resolution.

We also demonstrated via small-animal imaging that the 3-D CA imaging protocol and reconstruction methods worked reasonably well for the ex vivo rat heart containing a low-contrast and slowly varying distribution of radioactivity. This small-animal study also showed that the improvement made by dividing the object plane into four small regions in the projection model to account for the angular variations was effective and further enhanced the contrast in the reconstructed cross-sectional SPECT images. As compared to the commercial 5-pinhole reconstruction, our 3-D CA reconstruction resulted in less noise for identifying the myocardial perfusion defect (Fig. 11). Nevertheless, the image resolution remained suboptimal due to a number of inherent system limitations, such as the relatively poor photon detection efficiency of NaI and the small magnification factor of 2.6. The imaging system with the CA module mounted requires further calibration to account for certain imperfections of the rotation mechanism and imaging module, such as off-center rotation (rotation axis being off center) and camera alignment (the mask surface being un-parallel to the detector surface and/or the detector surface being un-parallel to the rotation axis), which was not done in this study. Such calibration would further improve the image reconstruction. Also, we envision that the current CA SPECT system can be greatly improved by using solid-state (such as CdZnTe) detectors with a smaller pixel size and by a new system configuration with a higher magnification factor.

## Conclusion

The simulations presented in this study showed that CA collimation yielded images with lower MSEs compared to those obtained with pinhole collimation in reconstructing a 2-D phantom with both hot and cold lesions embedded in a warm background. The small-animal imaging results showed a reasonably good 3-D reconstruction of the ex vivo rat heart with MI (visually a cold lesion) using CA collimation. These results demonstrate the feasibility of achieving high sensitivity and high resolution in near-field SPECT using CA collimation, and the advantage of CA in terms of CNR in imaging objects with a smooth distribution and cold lesion employing the MLEM reconstruction algorithms, as opposed to those derived based on conventional decoding methods.

The experimental data presented herein lays a foundation for the development of high-sensitivity and high-resolution SPECT imaging systems with near-field CA collimation, and opens up several avenues for future research. One of our research directions is to develop a flexible simulation tool that is suitable for CA imaging to avoid constantly dealing with non-ideal experimental set-ups and help us understand the characteristics of different system designs and imaging protocols. There is still room for improvement in the image reconstruction algorithms, where further optimization is desired for better image quality, faster computation and convergence, and more robust implementation. In addition, comparison with competing technologies, e.g., multi-pinhole collimations, is of great interest. Ultimately, as small-animal imaging finds more applications in biomedical research, we believe that it is valuable to explore ways to optimize the system and imaging scheme for small-animal studies.
